# Biomarkers immune monitoring technology primer: Immunoscore® Colon

**DOI:** 10.1186/s40425-016-0161-x

**Published:** 2016-09-20

**Authors:** Fabienne Hermitte

**Affiliations:** HalioDx, Marseille, France

## Description of the technology

The Immunoscore® assay for Colon Cancer (IS Colon) is the first standardized immune-based assay for the classification of cancer. It assesses the host immune response to Colon Cancer (CC) by measuring intra- and peri-tumoral T cell infiltration in formalin-fixed paraffin-embedded (FFPE) tissue sections.

For each tumor sample, 2 slides are stained using an automated immunohistochemistry (IHC) staining instrument (BenchMark XT, Ventana): one with CD3 and one with CD8 ready-to-use monoclonal antibodies (HalioDx). Detection is performed with the ultraView Universal DAB Detection Kit (Ventana), followed by counterstaining in order to visualize nuclei (Bluing Reagent, Ventana). Stained slides are then washed, dehydrated, mounted and coverslipped. Digital images of stained slides are obtained using a whole slide scanner (Nanozoomer XR, Hamamatsu), and analyzed by a software program (Immunoscore® Analyzer, HalioDx) if staining and image quality is validated by the operator.

One separate control slide with 3 external controls −1 negative tissue (placenta) and 2 positive (1 tissue: tonsil and a cell line pellet) - is processed identically in each IHC run, and allows monitoring of the staining and scanning steps.

### Image processing

Digital images are imported into the Immunoscore® Analyzer software and automatically processed for tissue detection (core of the tumor (CT), healthy non-epithelial tissue, and epithelium) and CD3 and CD8 positive lymphocytes quantification. The histotechnician reviews the region annotations and modifies them if required; the invasive margin (IM) is subsequently displayed automatically. Its width spans 360 μm into the healthy tissue and 360 μm into the tumor from the frontier between these two tissue types.

### Density and score calculation

After image processing validation by an independent reviewer, densities of CD3 and CD8 positive lymphocytes in the 2 regions of interest (ROIs) CT or IM regions are reported.

The best performing algorithm to compute the Immunoscore has been defined in the large international SITC -led retrospective validation study [1, 2] conducted on more than 3800 St I-III colon cancer patients, Briefly, for each marker (CD3 & CD8) and each zone (CT & IM), densities distributions have been established on the study training set; for each parameter of a tested sample (CD3, CD8, CT, IM), a percentile is derived from these distributions. An average percentile is calculated based on these 4 values. The Immunoscore® is reported as IS-0, 1 – 2 – 3 – 4 based on the following average percentile classes respectively: [0 %; 10 %] - [>10 %; 25 %] - [>25 %; 70 %] - [>70 %; 95 %] - [>95 %; 100 %].

### Reported prognostic information

Statistically significant prognostic groups have been defined in the SITC study [2], with the Immunoscore® categories 0 and 1 indicating a bad prognosis (high risk of relapse), while the Immunoscore® categories 3 and 4 indicate a good prognosis (low risk of relapse), and patients with an Immunoscore® 2 have an intermediate risk of relapse.

## Type of data obtained/readout

The Immunoscore® Analyzer generates a printable report. This report contains a capture of all quality control results as well as the analysis result.
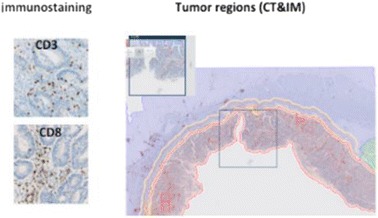


## Limitations of the approach

In order to get an accurate assessment of intra- and peri-tumoral T-cell infiltration, the tumor specimen must be selected from the block the most infiltrated by immune cells and must contain both the core of the tumor and the invasive margin. Pathologist review is mandatory to qualify the selection of the specimen according to those criteria prior to the testing.

The image analysis is a critical step in the process, and digital pathology expertise is required. The Immunoscore® assay is offered through HalioDx service laboratory and will be initially implemented in expert centers only.

Finally Immunoscore® optimal performance were validated on a specific platform combining validated instruments (autostainer and scanner) and qualified reagents. Future equivalency studies will be conducted to qualify additional platforms.

## Types of samples needed and special issues pertaining to samples

Starting material is a FFPE tissue block from a surgical section of colon cancer. The block must contain the core of the tumor (CT) and the invasive margin (IM) (tumor and healthy tissue present in the sample). Samples must have been fixed with 10 % buffered formalin. Slides must be prepared less than 4 months before testing; 2 slides are required (one for CD3, one for CD8) from adjacent 4 μm slices. Slices should be mounted on positively charged glass slides (Superfrost™ Plus or equivalent).

## Level of evidence

The role of adaptive immune response in controlling tumor progression in colorectal cancer was first evidenced by Galon et al. in 2006 [3]. In a comprehensive analysis of the tumor microenvironment, they showed that the type, density and location of immune cells within tumor regions predicted clinical outcome of the patients. In particular, the combined analysis of immune cells density in both tumor regions (CT and IM) was shown to improve the accuracy of survival prediction as compared to single-region analysis. It is based on those observations that the Immunoscore® was established and its prognostic power has been shown to surpass that of the conventional TNM staging and clinicopathological factors [3–6]. Notably, when Immunoscore® was applied on two large independent cohorts of early-stage colorectal cancer (*n* = 602), only 4.8 % of patients with a high Immunoscore relapsed after 5 years as opposed to 72 % of patients with a low IS indicating that these patients could potentially have benefited from adjuvant therapy.

Recent data in large cohorts of CRC patients further demonstrated the critical importance of IS over tumor-related features in the mechanism of dissemination to distant metastasis [7].

Immunoscore® was shown to be a stronger predictor of survival than microsatellite instability status [8]. Microsatellite instable (MSI) tumors often contain intra-epithelial T cells in response to the expression of neo-antigens on the cell surface and this probably contributes to the better prognosis of patients with MSI tumors. Although patients with IS-High tumors are overrepresented in the MSI group compared to the microsatellite stable (MSS) group, there is a substantial number of IS-High cases in the MSS group, indicating that there is a good number of MSS cases that are immunogenic. Patients with IS-High tumors had statistically significant prolonged survival than patients with IS-Low tumors despite their microsatellite status.

The last major Immunoscore® study conducted by the Immunoscore® worldwide consortium, and led by the Society for Immunotherapy of Cancer (SITC) involved 23 pathology centers from 17 countries. This international study including more than 3800 stage I/II/III colon cancer patients aimed at promoting the Immunoscore® in routine clinical setting [1,2]. Although CD45RO was one of the markers used for Immunoscore assessment in previous studies, because of background staining and loss of antigenicity in stored sections, it was agreed to employ the combination of the two easiest membrane stains, CD3 and CD8 in two regions (CT and IM) for validation in standard clinical practice [9]. The primary endpoint of the study was reached, with significantly longer TTR for patients classified as IS-High in the training set (HR = 0.41 (CI95 %, [0.28–0.61]; *p* < 0.0001) and two independent validation sets. Importantly, Immunoscore® discriminated a subgroup of high-risk stage II patients. Immunoscore® was also able to predict DFS and OS. Those major results, beyond further validating Immunoscore® as a strong prognostic marker also demonstrated that the assay was quantitative, reproducible and robust through this multicentric study.

Altogether, those results might lead the introduction of Immunoscore® into the cancer classification designated “TNM-I” (for TNM-Immune) and thereby help with therapeutic decision in clinical routine [9].

Immunoscore® Colon is available through HalioDx service laboratory, for research use only (RUO). By the end of the year, pathologists will have access to an in vitro diagnostic (IVD) assay in Europe, while a RUO solution will also be available in the rest of the World.

## Abbreviations

CC: Colon cancer
CRC: Colorectal cancer
CT: Core of the tumor
DAB: Diaminobenzidine
DFS: Disease-free-survival
DP: Digital pathology
DSS: Disease-specific-survival
FFPE: Formalin-fixed paraffin-embedded
IHC: Immunohistochemistry
IM: Invasive margin
INSERM: Institut national de la santé et de la recherche médicale
IS: Immunoscore
IVD: In vitro diagnostic
MSI: Microsatellite instable
MSS: Microsatellite stable
OS: Overall survival
QC: Quality control
ROI: Region of interest
RUO: Research use only
SITC: Society for immunotherapy of cancer
TNM: Tumor burden/ lymph nodes/ metastases

## References

[1] Galon J, Pagès F, Marincola FM, Angell HK, Thurin M, Lugli A (2012). Cancer classification using the immunoscore: a worldwide task force. J Transl Med.

[2] Galon J (2016). J Clin Oncol.

[3] Galon J, Costes A, Sanchez-Cabo F, Kirilovsky A, Mlecnik B, Lagorce-Pagès C (2006). Type, density, and location of immune cells within human colorectal tumors predict clinical outcome. Science.

[4] Galon J, Fridman WH, Pagès F (2007). The adaptive immunologic microenvironment in colorectal cancer: novel perspective. Cancer Res.

[5] Pagès F, Kirilovsky A, Mlecnik B, Asslaber M, Tosolini M, Bindea G (2009). In situ cytotoxic and memory T cells predict outcome in patients with early-stage colorectal cancer. J Clin Oncol.

[6] Mlecnik B, Tosolini M, Kirilovsky A, Berger A, Bindea G, Meatchi T (2011). Histopathologic-based prognostic factors of colorectal cancers are associated with the state of the local immune reaction. J Clin Oncol.

[7] Mlecnik B, Bindea G, Kirilovsky A, Angell HK, Obenauf AC, Tosolini M (2016). The tumor microenvironment and Immunoscore are critical determinants of dissemination to distant metastasis. Sci Transl Med.

[8] Mlecnik B, Bindea G, Angell HK, Maby P, Angelova M, Tougeron D (2016). Integrative analyses of colorectal cancer show immunoscore is a stronger predictor of patient survival than microsatellite instability. Immunity.

[9] Galon J, Mlecnik B, Bindea G, Angell HK, Berger A, Lagorce C (2014). Towards the introduction of the ‘Immunoscore’ in the classification of malignant tumours. J Pathol.

